# Wnt inhibitory factor 1 (WIF1) methylation and its association with clinical prognosis in patients with chondrosarcoma

**DOI:** 10.1038/s41598-017-01763-8

**Published:** 2017-05-08

**Authors:** Pei Liu, Jacson K. Shen, Francis J. Hornicek, Fuyun Liu, Zhenfeng Duan

**Affiliations:** 1 Sarcoma Biology Laboratory, Center for Sarcoma and Connective Tissue Oncology, Massachusetts General Hospital, Harvard Medical School, Boston, MA 02114 USA; 2 Department of Orthopaedics, The Third Affiliated Hospital of Zhengzhou University, Zhengzhou, Henan 450052 People’s Republic of China

## Abstract

Chondrosarcoma (CS) is a rare cancer, but it is the second most common primary malignant bone tumor and highly resistant to conventional chemotherapy and radiotherapy. Aberrant DNA methylation in the promoter CpG island of Wnt inhibitory factor 1 (WIF1) has been observed in different cancers. However, no studies have shown the relationship between WIF1 methylation and CS. In this study, we found promoter methylated WIF1 in both CS cell lines (CS-1 and SW1353) and tumor tissues. Western blot analysis confirmed loss WIF1 expression and activation of Wnt pathway proteins (Wnt5a/b, LRP6, and Dvl2). We subsequently examined the correlation between levels of WIF1 methylation and overall survival (OS) and progression-free survival (PFS) in CS patient samples with a follow-up spanning 234 months (mean: 57.6 months). Kaplan-Meier survival curves and log-rank tests revealed that high levels of WIF1 methylation were associated with lower OS and PFS rates (p < 0.05). Multivariate Cox hazard analysis suggested that detection of high level methylation of WIF1 could be an independent prognostic factor in OS and PFS. In conclusion, we found that WIF1 is epigenetically silenced via promoter DNA methylation in CS and propose that WIF1 methylation may serve as a potential prognostic marker for patients with CS.

## Introduction

Chondrosarcoma (CS) is a heterogeneous subtype of malignant cartilage forming tumor. It is a rare cancer in humans and is diagnosed in approximately 600 patients per year in the United States. CS is the second most common primary bone cancer after osteosarcoma^1, 2^. CS accounts for more than 20% of all primary bone malignancies and affects people at any age with a predilection for proximal femur and pelvic sites^3–5^. Pathological identification and radiographic imaging remain important diagnostic tools in the clinical management of patients with CS. However, the reliability of diagnosis by the current subjective imaging and histological criteria has been controversial^6^. CS, especially conventional CS, is notoriously resistant to both chemotherapy and radiation treatment^7^. Thus, surgical resection remains the primary treatment for localized lesions and radiotherapy is limited to the definitive treatment of inoperable micro-lesions and in the palliation of local symptoms^6^. The outcome of patients with CS is relatively poor largely due to the potent capacity for local invasion and distant metastasis^8, 9^. Five-year survival rates of patients with dedifferentiated CS are less than 20% as a result of early disseminated metastases^6, 10–12^. Therefore, there is an urgent need to develop novel strategies for diagnosis, treatment, and prognosis of CS to improve outcomes for patients.

Recently, aberrant DNA methylation and epigenetic changes have been found to play a pivotal role in the pathogenesis of human malignancies. Hypermethylation mostly presents at the promoter CpG island of tumor suppressor genes and inactivates gene expression, while hypomethylation commonly affects repetitive DNA sequences and/or gene regulatory regions^13^. Accumulating data has shown that DNA methylation of tumor-related genes may serve as biomarkers to indicate the diagnosis and/or prognosis of human cancers, including CS^14^. For example, methylation of tumor suppressor gene Runt-related transcription factor 3 (RUNX3) has been reported as a potential prognostic indicator for CS^15, 16^. Recently, methylation of Wnt inhibitory factor 1 (WIF1) has also been found in different cancers, including in lung cancer, gastric cancer, and osteosarcoma^17–21^. However, no studies have shown the relationship between WIF1 methylation and CS.

WIF1 is a lipid-binding protein coding gene that binds to Wnt proteins, preventing them from passing signals to the cell. WIF1 is believed to function as a tumor suppressor gene by disrupting Wnt signaling, including the Wnt canonical pathway that regulates gene transcription, and could activate various downstream oncogenes. Wnt proteins comprise a large family of secreted cysteine-rich glycoproteins and have been found to play critical roles in the development and progression of human cancers^22, 23^. Each member of the Wnt family is defined by its amino acid sequence rather than the functions of its protein. Wnt has been associated with a number of different activities and downstream signaling pathways. The majority of effort in this field of cancer research has been focused on β-catenin or canonical-dependent Wnt signaling. Normally, Wnt ligands bind to the frizzled and low-density lipoprotein receptor related protein-5/6 (LRP-5/6) and subsequently activate the intracellular protein, Dishevelled (Dvl). Activated Dvl leads to the repression of glycogen synthase kinase 3β (GSK-3β), resulting in the disruption of the multi-protein complex, which is involved in the degradation of β-catenin. This complex is comprised of GSK-3β, adenomatous polyposis coli (APC), and axin^23^. Hence, β-catenin accumulates in the cytoplasm and translocates to the nucleus, where in cooperation with members of the T cell factor/lymphoid enhancer factor (TCF/LEF) family, subsequently activates several oncogenes, including c-Myc, cyclin D1, metalloproteinases, and c-Met^21, 23^. Nevertheless, Wnt antagonists, including WIF1, are able to collapse this pathway by inhibiting the binding of Wnt ligands to receptor complexes, followed by β-catenin phosphorylation and degradation, and finally blocking the TCF/LEF transcription of a wide range of oncogenes, thus preventing tumorogenesis^21, 23^. Dysregulation of Wnt signaling has been observed in various bone malignancies, including osteosarcoma, Ewing sarcoma, and CS^20, 21, 24–28^. Wnt signaling is tightly regulated by either directly binding with Wnt ligands, WIF1, or indirectly binding to Wnt receptors, such as Dickkopf (Dkk) family proteins^21^. Elevated DKK levels have been found to be significantly correlated with the expression of β-catenin in CS^29^. Targeting the Wnt/β-catenin pathway has been proposed as a potential therapeutic treatment for sarcoma. However, the relationship between the epigenetic status of WIF1 and the activation and expression of Wnt in CS is unknown.

In this study, our primary aim was to assess the status of WIF1 DNA methylation in CS and to further examine the correlation between levels of WIF1 methylation and clinical prognosis of CS. Protein expression levels of WIF1 and members of the Wnt family were also measured to assess the potential tumor suppressor role of methylated WIF1 in the Wnt pathway in CS.

## Materials and Methods

### Cell lines and cell culture

The human CS cell line SW1353 was purchased from American Type Tissue Collection (Rockville, MD). The human CS cell line CS-1 was established in our laboratory as previously reported^30–32^. These two cell lines were cultured in RPMI 1640 medium (Invitrogen) supplemented with 10% fetal bovine serum, 100 units/ml penicillin, and 100 μg/ml streptomycin (Invitrogen). Cells were incubated at 37 °C in 5% CO_2_-95% air atmosphere. DNA extraction was performed when cells were near-confluent monolayers and collected by using trypsin-EDTA solution.

### Demethylation treatment

CS cell lines SW1353 and CS-1, seeded in 6-well plates at 5 × 10^5^ cells per well, were allowed to attach overnight and then treated with demethylation drug 5-Aza-2′-deoxycytidine (5-Aza-dC, Sigma-Aldrich, MO, USA) dissolved in RPMI 1640 medium at 1, 5, and 10 μmol/L for 3 days. During the course of treatment, medium was changed every day with the same concentration of 5-Aza-dC. At the end of the treatment period, cells were washed twice with PBS and harvested for western blot analysis.

### Patient specimens and clinical data

The study and the consent of the informed patients were approved by the Partners Human Research Committee (number: 2007P-002464). All patients were informed and provided written consent that their information and the use of tissue samples stored in the databases of MGH Sarcoma Tumor Bank would be utilized for research. The information or images contained in the manuscript could not lead to identification of any study participant. All methods described in this manuscript were performed in accordance with the relevant guidelines and regulations by our institution. A retrospective study of 45 tumor specimens from 40 individual CS patients was evaluated by using the MGH Cancer Registry and the Orthopedic Oncology database. Histologic subtype and grade were scored by pathologists at MGH. The following patient data was collected: age, gender, histologic subtype, tumor grade, tumor location, metastatic status, recurrent status, disease status, and date of death (if applicable). The clinical data of CS patients are shown in Table 1.

**Table 1:** Status of WIF1 methylation in CS tissue and their relations to clinical parameters.

Clinical parameters	Total *N*	WIF1 methylation level (%) [mean ± SE]	*P* value
All cases	43	46.61 ± 6.09	
Age (years)			0.072
≤45	21	36.25 ± 8.77	
>45	22	56.49 ± 8.09	
Gender			0.895
Male	30	45.21 ± 7.28	
Female	13	49.83 ± 11.50	
Histologic Subtype			0.571
Hyaline and/or Myxoid	21	45.32 ± 9.17	
Dedifferentiated	12	57.10 ± 9.56	
Mesenchymal	3	29.08 ± 29.04	
NOS	5	51.80 ± 21.54	
Grade			0.195
1	10	28.68 ± 12.79	
2	23	46.99 ± 8.59	
3	9	68.44 ± 9.62	
Metastatic			0.358
Present	10	55.69 ± 12.86	
Absent	33	43.86 ± 6.95	
Recurrent			0.267
Present	12	36.16 ± 12.70	
Absent	31	50.65 ± 6.87	
Location			0.237
Limbs	25	40.33 ± 8.00	
Trunk	18	55.32 ± 9.26	

SE represents standard error; NOS represents not otherwise specified.

### DNA isolation

Extraction of DNA from CS cell lines SW1353, CS-1, and tumor tissues were performed using QIAamp DNA Micro kit (Qiagen, Valencia, CA). The extraction was carried out according to the manufacturer’s instructions. Briefly, CS tumor tissue samples or cell pellets from cultured cell lines, approximately 8 mg in weight, were transferred to a 1.5 mL microcentrifuge tube, and 180 µL of buffer ATL (Qiagen) were added immediately. After equilibrating to room temperature (25 °C), 20 µL of proteinase K was added and cell were vortexed for 15 seconds. The sample tube was incubated at 56 °C overnight until the sample was completely lysed. The next day, 200 µL buffer AL (Qiagen) was added and cells were again vortexed for 15 seconds. Subsequently, 200 µL of ethanol (96–100%) were added. The mixture obtained was loaded on a QIAampMiniElute spin column provided by the kit and washed with AW1 buffer followed by AW2 buffer (Qiagen). DNA was eluted with 60 µL of buffer AE (Qiagen) and preserved at −20 °C until use.

### Quantitative real-time PCR of WIF1 promoter methylation

The WIF1 promoter methylation levels in CS cell lines and tumor tissues were determined by MethylScreen technology using the EpiTect Methyl II PCR Array (Qiagen). This study in CS cell lines used a 22 tumor suppressor gene panel that included WIF1 (Supplementary Table 1). The method employed by the EpiTect Methyl II PCR System can distinguish methylated from unmethylated sequences through methylation-sensitive (Ms) and/or methylation-dependent (Md) restriction enzymes cleaving target CpG islands or not, rather than converting unmethylated CpG sites by bisulfite treatment. Following digestion, the remaining DNA of each digestion was quantified by real-time PCR. The relative fractions of methylated and unmethylated DNA were subsequently calculated by comparing the amount in each reaction with that of a mock (Mo, no enzymes added) reaction. Briefly, 1 μg or 125 ng genomic DNA was equally separated into four reactions, which were subsequently subjected to mock digestion, digestion of Ms or Md restriction endonuclease individually, or both (Msd). The amount of target promoter sequence in each reaction was quantified by real-time PCR using primers with SYBR Green ROX Mastermix (Qiagen) and analyzed with a StepOnePlus Real-time PCR System (Applied Biosystems); all primers used in procedure are purchased from Qiagen. PCR cycling conditions were described as follows: 95 °C for 10 min, 3 cycles of 99 °C for 30 sec, 72 °C for 1 min, 40 cycles of 97 °C for 15 sec, 72 °C for 1 min with real-time signal acquisition. The melting curve was performed using the default program on the instrument after the cycling program.

### WIF1 methylation analysis

Upon completion of real-time PCR, C_T_ values were obtained by setting the threshold values and baseline as follows: 0.2 (threshold cell lines), 0.02 (threshold tissues), and 2–15 (baseline). The identical threshold and baseline are set for comparing multiple plates between CS cell lines or among CS tissue samples. This was repeated if the C_T_ value did not pass the data quality control, which required the C_T_ value of mock digest to be within the range of 18 to 27 cycles and the difference in C_T_ value between Msd and mock to be greater than 3(ΔC_T_ [Msd − mock] > 3). Subsequently, the methylation status was calculated by ΔC_T_ method following manufacturer’s instruction. Due to the inversely proportional relationship between threshold cycle and the amount of input DNA, and due to the doubling of PCR product with every cycle in the exponential phase of the reaction, the initial DNA amount in each digest before PCR was expressed as:

1 $${{\rm{C}}}_{{\rm{Mo}}}={2}^{-{\rm{Ct}}({\rm{Mo}})};{{\rm{C}}}_{{\rm{Ms}}}={2}^{-{\rm{Ct}}({\rm{Ms}})};{{\rm{C}}}_{{\rm{Md}}}={2}^{-{\rm{Ct}}({\rm{Md}})};{{\rm{C}}}_{{\rm{Msd}}}={2}^{-{\rm{Ct}}({\rm{Msd}})}$$

The fraction of DNA in each digest was calculated by normalizing the DNA amount to the amount of digestible DNA. The amount of digestible DNA was equal to the total amount of DNA (determined from the mock digest) minus the amount of DNA resistant to DNA digestion (determined from the double digest).

Hypermethylated (HM) DNA Fraction:

2 $${{\rm{F}}}_{{\rm{HM}}}=\frac{{{\rm{C}}}_{{\rm{Ms}}}}{{{\rm{C}}}_{{\rm{Mo}}}-{{\rm{C}}}_{{\rm{Msd}}}}=\frac{{2}^{-{\rm{Ct}}({\rm{Ms}})}}{{2}^{-{\rm{Ct}}({\rm{Mo}})}-{2}^{-{\rm{Ct}}({\rm{Msd}})}}$$

Unmethylated (UM) DNA Fraction:

3 $${{\rm{F}}}_{{\rm{UM}}}=\frac{{{\rm{C}}}_{{\rm{Md}}}}{{{\rm{C}}}_{{\rm{Mo}}}-{{\rm{C}}}_{{\rm{Msd}}}}=\frac{{2}^{-{\rm{Ct}}({\rm{Md}})}}{{2}^{-{\rm{Ct}}({\rm{Mo}})}-{2}^{-{\rm{Ct}}({\rm{Msd}})}}$$

Intermediately methylated (IM) DNA fraction:

4 $${{\rm{F}}}_{{\rm{IM}}}=1-{{\rm{F}}}_{{\rm{HM}}}-{{\rm{F}}}_{{\rm{UM}}}$$

Methylated (M) DNA fraction:

5 $${{\rm{F}}}_{{\rm{M}}}={{\rm{F}}}_{{\rm{HM}}}+{{\rm{F}}}_{{\rm{IM}}}$$

Results of gene methylation were presented as the percentage unmethylated and methylated fraction of input DNA; methylated indicates the fraction of input genomic DNA containing two or more methylated CpG sites in the promoter of a targeted gene.

### Western blot

Total protein was isolated from CS-1 and SW1353 cells with 1 × RIPA lysis buffer (Upstate Biotechnology, Charlottesville, VA) supplemented with complete protease inhibitor cocktail tablets (Roche Applied Science, IN, USA). Protein concentrations were examined by Protein Assay Reagents (Bio-Rad, Hercules, USA). Equal amounts of denatured proteins were separated by NuPAGE® 4–12% Bis-Tris Gel (Life Technologies) and transferred onto a nitrocellulose membrane (Bio-Rad). After blocking in 5% non-fat milk for two hours, the membranes were incubated with specific primary antibodies WIF1 (Cell Signaling Technology, catalog number: 5652, 1:1000 dilution), Wnt5a/b (Cell Signaling Technology, catalog number: 2530, 1:1000 dilution), LRP6 (Cell Signaling Technology, catalog number: 3395, 1:1000 dilution), Dvl2 (Cell Signaling Technology, catalog number: 3224, 1:1000 dilution), and β-Actin (Sigma-Aldrich, dilution 1:2000) at 4 °C overnight. The membranes were further probed with respective secondary antibodies (LI-COR Biosciences, NE, USA), and scanned by Odyssey® CLx equipment (LI-COR Biosciences, NE, USA) to detect the bands and the density.

### Statistical analysis

All statistical analyses were carried out using SPSS 17.0 software (Chicago, IL). We confirmed continuous variables were not within normal distribution by using the Shapiro-Wilk test. Then the Wilcoxon analysis and Kruskal-Wallis test were used to compare methylation levels between groups, sorted by age, gender, metastatic, recurrent, grade, histologic subtype, and location. Kaplan-Meier survival curves were generated to determine the relationship between the methylation levels of WIF1 and patient prognosis, and the log-rank test was applied to detect the difference between survival curves. Cox proportional hazard regression model was used to estimate the effect on survival outcomes of histologic subtype, grade, metastatic status, recurrent situation, tumor location, and DNA methylation level. Multivariate analysis was performed by the Cox proportional hazard method with the inclusion of all the variables taken into account for univariate analysis. Overall survival (OS) time was calculated from the date of tumor diagnosis to the date of death or last follow-up. Progression-free survival (PFS) time was calculated from the date of tumor diagnosis to the date of death or occurring metastasis and/or recurrence. All p-values were two-sided and p < 0.05 was considered as statistically significant.

## Results

### Identification of WIF1 promoter methylation in CS cell lines

**Figure 1:**
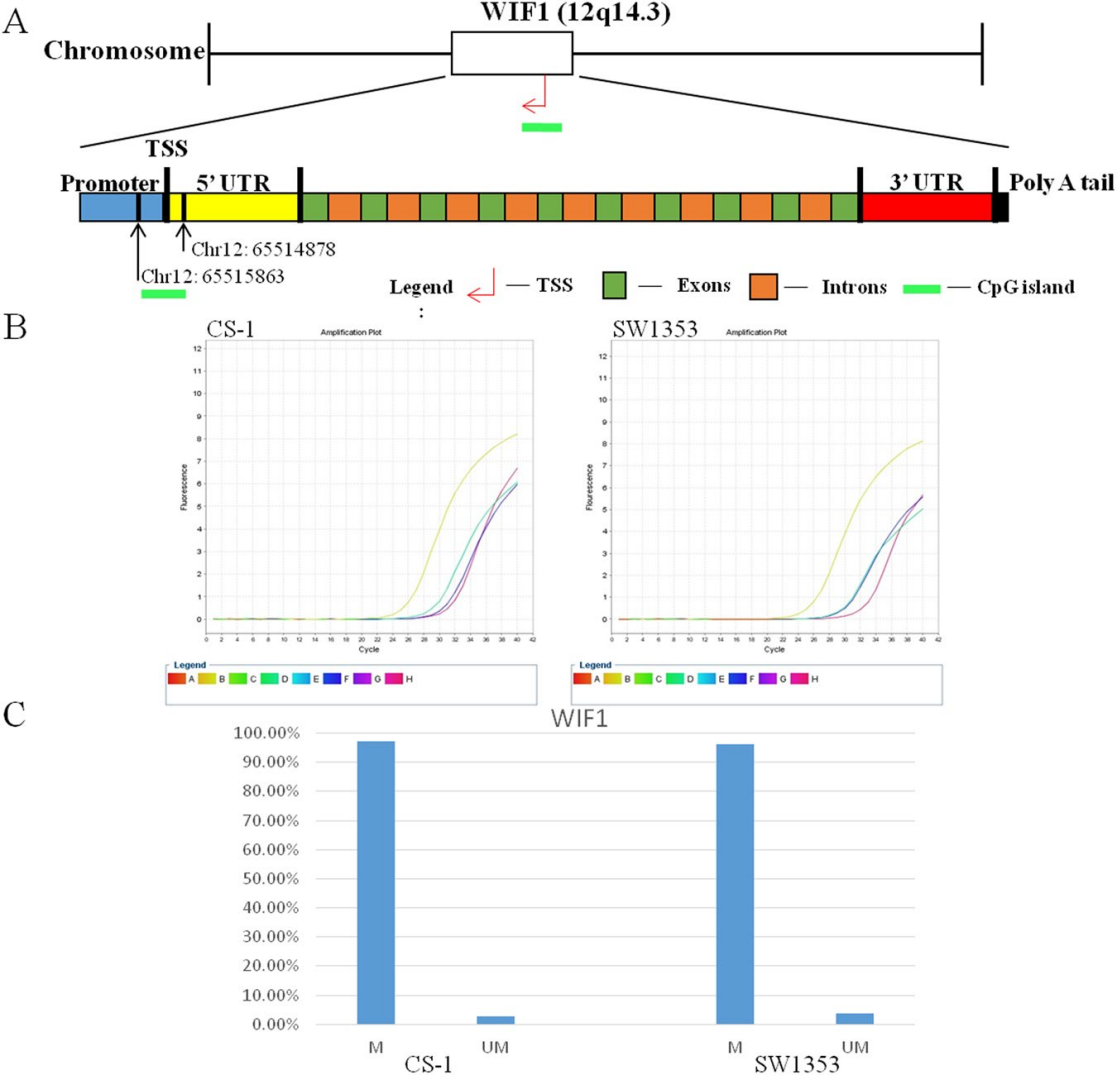
(**A**) The location of the CpG island in the WIF1 promoter. The target CpG island is located in Chr12: 65514878–65515863. (**B**) Representative amplification plots from real-time PCR of WIF1 in CS cell lines. Yellow curve (Legend B) indicates group Mo, which was subjected to no enzyme and represents the total amount of input DNA; Green curve (Legend D) indicates group Ms, which was subjected to methylation-sensitive enzyme and represents DNA in which all CpG sites are methylated; Blue curve (Legend F) indicates group Md, which was subjected to methylation-dependent enzyme and represents unmethylated DNA; Pink curve (Legend H) indicates group Msd, which was subjected to both methylation-sensitive and methylation-dependent enzyme and represents DNA refractory to enzyme digestion (background signal). The X-axis shows the cycle number of real-time PCR, and Y-axis shows fluorescence intensity. (**C**) Methylation profile of WIF1 in CS cell lines. The methylation event of the promoter in WIF1 is high frequency in CS cell lines CS-1 (M, 97.33%; UM, 2.67%) and SW1353 (M, 96.22%; UM, 3.78%). M represents methylation, UM represents unmethylated.

We examined the methylation levels of WIF1 promoter (Fig. 1A) in CS cell lines CS-1 and SW1353. The WIF1 gene was chosen for this study because of its known involvement in regulating tumorogenesis in various cancers, but had not yet been studied in CS (Supplementary Table 1). Representative amplification curves of WIF1 in two cell lines are showed in Fig. 1B. We found 97.33% of WIF1 promoters were methylated in CS-1 and 96.22% in SW1353, respectively (Fig. 1C). The methylation levels of 22 genes range from 0.51% to 98.81% in CS-1 and from 0.05% to 99.61% in SW1353.

### DNA methylation can silence WIF1 expression and increase Wnt signaling in human CS cell lines

**Figure 2:**
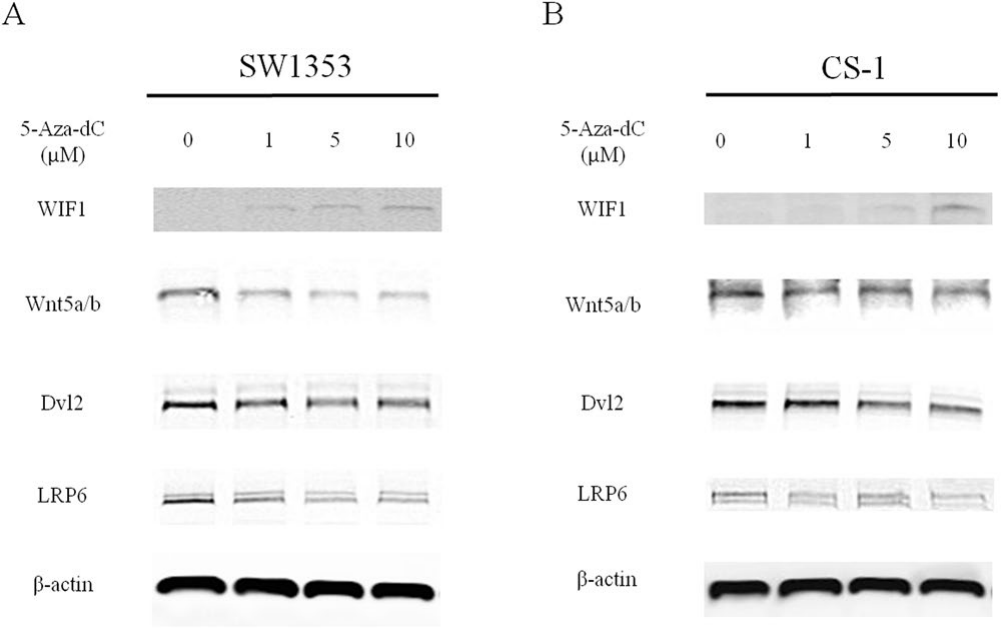
Epigenetic silencing of WIF1 derepresses Wnt signaling. (**A**) In CS cell line SW1353, Western blot analysis showed a trend induction of WIF1 expression after treatment with 1, 5, 10 μM 5-Aza-dC for 3 days. Wnt components including Wnt5a/b, Dvl2, and LRP6 were simultaneously suppressed with gradually restored WIF1 protein level. (**B**) In CS cell line CS-1, WIF1 was downregulated but re-expressed after reversion of DNA methylation. A notable repression of Wnt signaling (Wnt5a/b, Dvl2, and LRP6) was exhibited after the activation of WIF1. Western blot images were cropped to improve the clarity and conciseness of the result; samples derive from the same blots were processed in parallel.

Since WIF1 showed promoter methylation in CS cell lines, Western blot analysis was used to further determine the protein expression of WIF1. As expected, WIF1 exhibited loss of expression in both CS cell lines CS-1 and SW1353 (Fig. 2, Supplementary Figure S1). After treatment with methylation inhibitor 5-Aza-dC for the demethylation process, the expression of WIF1 was restored (Fig. 2, Supplementary Figure S1). Quantitative analysis of Western blot data showed that 5-Aza-dC enhanced WIF1 expression in a dose-dependent manner. In addition, we further analyzed WIF1 mediated Wnt signaling pathways in CS. Results demonstrated that demethylation of WIF1 led to a decreased expression of Wnt5a/b, LRP6, and Dvl2 in our CS cell lines (Fig. 2, Supplementary Figure S2). Taken together, these results suggest that DNA methylation of WIF1 can activate the Wnt pathway in CS.

### WIF1 promoter methylation level in CS patient tissues and its association with clinical data

**Figure 3:**
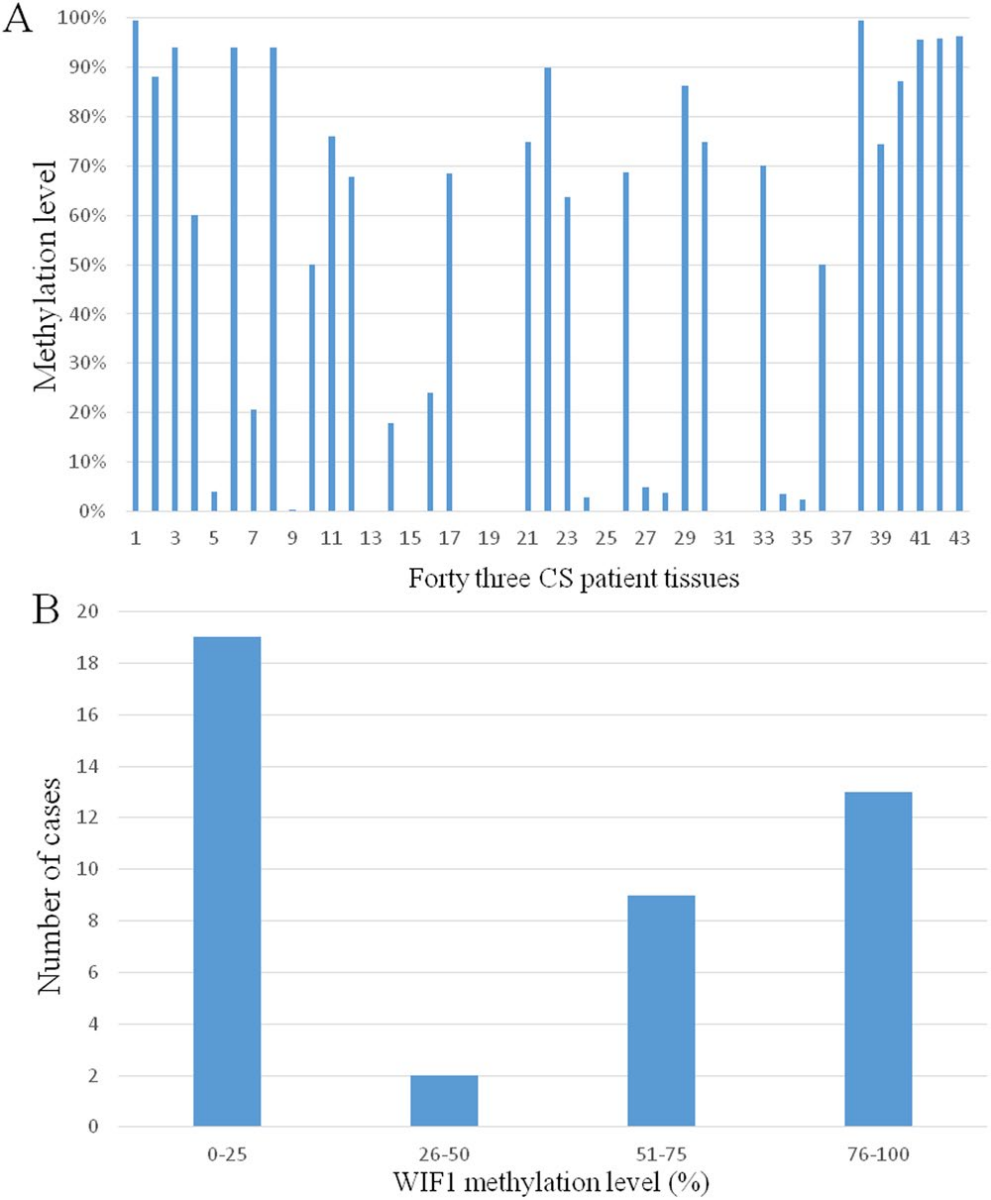
Methylation level of WIF1 in CS patient tissues. (**A**) Methylation levels of WIF1 in each specimen are presented. (**B**) Distributions are shown for 43 CS tumor samples.

We further examined the methylation levels of WIF1 in 45 tumor specimens of patients with CS. Two of the 45 cases were excluded since their C_T_ values did not pass quality control (after repeating three times). The WIF1 methylation level in 43 CS tissue samples were distributed as follows: mean 46.61; median 60.13; standard deviation (SD) 39.92; range 0.00–99.54 (Fig. 3A). Methylation of promoter CpG islands was detected in 19 cases (44%) for levels between 0% to 25%, two cases (5%) for 26% to 50%, nine cases (21%) for 51% to 75%, and 13 cases (30%) for 76% to 100% (Fig. 3B). Median age at diagnosis was 47 years (range 21–80), and sex ratio (male/female) was 2.3:1 (30 vs. 13). Ten cases (23%) had metastatic disease and 12 cases (39%) were recurrent. We then analyzed the relationship between the WIF1 methylation level and various clinical variables. Among the cases classified by histologic subtype, one case ‘Diffuse large cell Lymphoma’ and one case ‘Juxtacortical’ were excluded since the case number was too small for statistical analysis. Across the cases classified by grade, one case was excluded because the grade of the tumor was unavailable. A weak correlation between WIF1 methylation level and age (*p* = 0.072) was observed but was not statistically significant (Table 1). Importantly, none of the clinical parameters (gender, histologic subtype, grade, metastatic status, recurrent status, and location) of CS were significantly correlated with WIF1 methylation level (Table 1).

### WIF1 methylation level and patient survival

**Figure 4:**
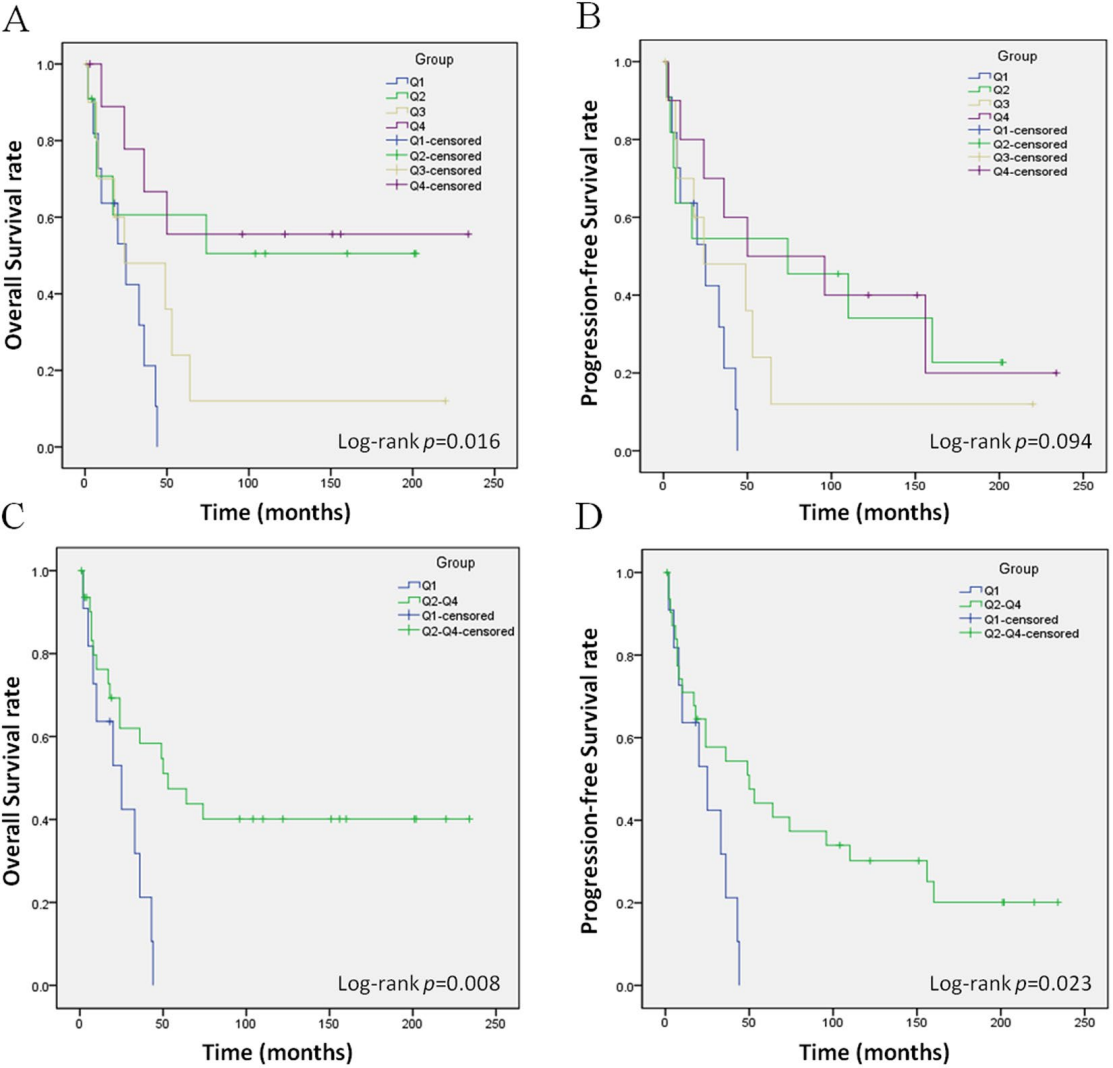
Kaplan-Meier curves of overall survival and progression-free survival of different quartiles of WIF1 methylation level in CS. (**A**) Kaplan-Meier curves of OS showed a significant difference across the Q1, Q2, Q3, and Q4 groups (*p* = 0.016). (**B**) Kaplan-Meier curves of PFS showed a weak difference among these four groups, but was not significant (*p* = 0.094). (**C**) OS of Q1 group was significantly shorter than the Q2–4 group (*p* = 0.008). (**D**) PFS of Q1 group was significantly shorter than the Q2–4 group (*p* = 0.023). Q1 represents the “high-methylation-level group” and Q2, Q3, and Q4 collectively represent the “low-methylation-level group”.

We assessed the association of WIF1 methylation level with survival outcomes. Among 43 cases of CS, 17 of them (40%) had died of disease within a median follow-up period of 25 months; mean period of total follow-up time was 57.63 months (range from 1 to 234 months). Based on the methylation level of patient samples, we categorized all cases into four quartiles as previous describe (Q1: ≥87.17%; Q2: 60.13–87.17%; Q3: 2.49–60.13%; Q4: <2.49%)^33, 34^. The Kaplan-Meier analysis showed that overall survival was significantly different among the four groups (log-rank *p* < 0.05); however, progression-free survival was not significantly different across the four groups (log-rank *p* = 0.094) (Fig. 4). Q1 was defined as the “high-methylation-level group” and Q2, Q3, and Q4 were combined into the “low-methylation-level group”^33^. The survival time of the “high-methylation-level group” was significantly shorter than that of the “low-methylation-level group” for overall survival and progression-free survival (log-rank *p* < 0.05) (Fig. 4). The median survival times for overall and progression-free in each group are shown in Table 2.

**Table 2:** Medians (months) for OS and PFS.

Group	Overall Survival			Progression-free Survival		
	Estimate	SE	CI	Estimate	SE	CI
Q1	25.000	11.034	3.373–46.627	25.000	11.034	3.373–46.627
Q2–4	53.000	18.081	17.561–88.439	50.000	19.402	11.972–88.028
Overall	36.000	11.322	13.810–58.190	36.000	11.604	13.255–58.745

SE represents standard error; CI represents confidence interval; Q1 represents the “high-methylation-level group”; Q2–4 represent the “low-methylation-level group”.

Next, we confirmed the above observations using univariate Cox analysis (Table 3, Table 4). In addition, histologic subtype, and metastatic status were found to be significantly associated with patient survival for overall survival (*p* < 0.05). In further study of multivariate Cox analysis, we found that the WIF1 methylation level was an independent prognostic factor in CS. Overall survival and progression-free survival were significantly shorter in the “high-methylation-level group” than in the “low-methylation-level group” (*p* < 0.05). The hazard rates (HR) for overall survival and progression-free survival were 2.893 (95%CI: 1.167–7.170) and 2.399 (95%CI: 1.020–5.641), respectively. Moreover, histologic subtype and metastatic status were found to be significantly related with patient survival for overall survival and histologic subtype was also significantly associated with patient outcome for progression-free survival (Table 3, Table 4).

**Table 3:** Univariate and multivariate analysis of overall survival by Cox proportional hazard regression model.

Parameters	Univariate analysis			Multivariate analysis		
	HR	CI	*P* value	HR	CI	*P* value
Age (discrete variable)	1.023	0.997–1.049	0.078	…	…	…
Gender (female as reference)	1.563	0.658–3.710	0.312	…	…	…
WIF1 methylation level (Q2–4 as reference)	2.980	1.268–7.003	0.012	2.893	1.167–7.170	0.022*
Histologic Subtype (Hyaline and/or Myxoid as reference)	…	…	0.007	…	…	0.002*
NOS	1.524	0.427–5.446	0.517	0.921	0.243–3.487	0.903
Dedifferentiated	4.753	1.930–11.705	0.001	5.881	2.277–15.187	0.000*
Mesenchymal	1.188	0.265–5.334	0.822	1.052	0.231–4.779	0.948
Grade (1 as reference)	…	…	0.111	…	…	…
2	2.164	0.781–5.999	0.138	…	…	…
3	3.431	1.081–10.887	0.036	…	…	…
Metastatic (Absent as refernece)	2.972	1.245–7.094	0.014	2.926	1.163–7.359	0.023*
Recurrent (Absent as reference)	0.623	0.262–1.482	0.284	…	…	…
Location (Limbs as reference)	1.141	0.533–2.444	0.734	…	…	…

… represents “data not available”; * represents *p* < 0.05; Abbreviations: HR, hazard ratio; CI, confidence interval; NOS, not otherwise specified.

**Table 4:** Univariate and multivariate analysis of progression-free survival by cox proportional hazards regression model.

Parameters	Univariate analysis			Multivariate analysis		
	HR	CI	*P* value	HR	CI	*P* value
Age (discrete variable)	1.019	0.996–1.042	0.110	…	…	…
Gender (female as reference)	1.317	0.623–2.786	0.471	…	…	…
WIF1 methylation level (Q2–4 as reference)	2.492	1.093–5.682	0.030	2.399	1.020–5.641	0.045*
Histologic Subtype (Hyaline and/or Myxoid as reference)	…	…	0.047	…	…	0.048*
NOS	2.070	0.747–5.738	0.162	1.622	0.564–4.666	0.369
Dedifferentiated	3.110	1.348–7.171	0.008	3.213	1.384–7.460	0.007*
Mesenchymal	0.944	0.216–4.132	0.939	0.895	0.204–3.934	0.884
Grade (1 as reference)	…	…	0.135	…	…	…
2	2.466	0.982–6.195	0.055	…	…	…
3	2.531	0.841–7.613	0.098	…	…	…
Location (Limbs as reference)	1.284	0.642–2.565	0.480	…	…	…

… represents “data not available”; * represents *P* < 0.05; Abbreviations: HR, hazard ratio; CI, confidence interval; NOS, not otherwise specified.

## Discussion

We have demonstrated for the first time WIF1 promoter methylation in CS cell lines CS-1 and SW1353. Western blot results showed that there was a silencing of WIF1 in CS cells. We also observed an upregulation of WIF1 expression after exposure to a demethylation agent. Our data suggests that WIF1 promoter methylation is an important epigenetic mechanism leading to WIF1 downregulation in CS. In osteosarcoma, WIF1 silencing due to promoter methylation has been recognized to facilitate tumor proliferation, migration, and lung metastasis *in vivo* and *in vitro* ^20, 21^. Considering this, we assume that aberrant methylation of WIF1 is a vital factor affecting Wnt function and downstream signaling in CS.

Previous reports have demonstrated that activation of the Wnt pathway exists in CS cells and primary CS tissue^27–29^. Regarding the close relationship between Wnt signaling and WIF1, we performed further experiments to verify whether the Wnt pathway was involved in the inhibitory effect of WIF1.

LRP6, an indispensable co-receptor for the Wnt signaling pathway, has been found highly expressed in several human cancer cell lines and human malignant tissues^35^. In agreement with this report, our data showed that LRP6 was highly expressed in CS cells. Importantly, LRP6 was simultaneously reduced with gradual restoration of WIF1 expression. Moreover, recent work reported that Dvl2 was overexpressed in prostate cancer cell lines and tissues and its silencing by RNAi can reduce cell proliferation and migration^36^. Likewise, we demonstrated an elevated protein level of Dvl2 in CS cell lines, but a diminished level after WIF1 recovery. Another Wnt component, Wnt5a/b, was found to be overexpressed and involved in the migration of Ewing sarcoma^37^. Similarly, there is also a notable expression of Wnt5a/b and an inverse correlation between the expression of Wnt5a/b and WIF1. Taken together, our results support the hypothesis that WIF1 restoration could attenuate the activity of Wnt pathway and impedes the transmission of Wnt signal.

Recently, preclinical work has focused on targeting Wnt signaling as a potential therapy. For example, LRP6 knockdown by shRNA in breast cancer cells can reduce cell proliferation and administration of LRP6 inhibitor, Mesd, is able to suppress tumor growth *in vivo* ^38^. Overexpression of Wnt5a in melanoma correlates with chemotherapeutic resistance^39^. In this regard, a novel therapeutic strategy could be developed to inhibit Wnt components, including LRP6, Dvl2, and Wnt5a/b through restoration of WIF1 expression.

The prognosis of CS has been related to various clinical parameters, such as histological grade, age, tumor size, and positive/negative surgery margin. Biomolecular markers, including RUNX3 promoter methylation, have also been associated with patient outcome^15, 40, 41^. It has also been shown that activation of Wnt signaling is associated with negative CS patient outcome^42^. However, the relationship between WIF1 methylation level and the prognosis of CS has not been demonstrated. Our data indicates that patients with higher WIF1 methylation levels have poorer overall survival and progression-free survival rates as compared with the WIF1 low-level group. This suggests that the WIF1 methylation level could be an independent prognostic factor for overall and progression-free survival. These findings explore a new prognostic marker in patients with CS. Additionally, histologic subtype, especially dedifferentiated CS, was also shown to contribute to a poorer outcome with the highest hazard rate compared with other factors. This is consistent with previous studies and clinical experience^6, 11^. Of note, regarding the limited publications of CS that are mainly composed of case reports or small case series, we found few studies that included more than 40 patients with relative long term follow-up data^41, 43–45^. For example, one methylation study focused on RUNX3 in CS patients with a total follow-up time of 5 years and an epigenetics study on microRNA-494 in CS patients again with a 5 year follow-up^8, 15^.

In a 44 case lung cancer study, WIF1 methylated patients had an inferior overall survival and multivariate analysis revealed WIF1 methylation was an independent prognostic factor in overall survival^46^. Interestingly, methylation of WIF1 has been demonstrated as an independent outcome predictor with a negative impact on patient prognosis in different cancers, including leukemia, lung cancer, breast cancer, and colon cancer^47–50^. Taken together, WIF1 methylation may be a common event in malignancies, and anti-methylation of WIF1 may improve patient survival and serve as a potential treatment in cancers, including in CS. Recent work has provided preclinical evidence that isoliquiritigenin treatment can prevent breast cancer initiation and progression through WIF1 demethylation^51^. In human non-small cell lung cancer, norcantharidin treatment was found to inhibit tumor cell proliferation and invasion by promoting WIF1 demethylation^52^. This is attractive as it may expand the activity of these agents in clinic. In this regard, it sheds novel light on selectively reversing epigenetic changes of WIF1 for CS prevention and a unique targeted strategy could emerge. Thus, although the mechanism of WIF1 methylation need to be further clarified, our work may contribute to an improvement in survival rate for patients with CS.

We next explored the WIF1 methylation level in CS patient tissues and analyzed the potential association of WIF1 methylation with other clinical parameters by performing statistical analysis. However, no significant associations were found. A larger scale study may be necessary to uncover any associations of WIF1 methylation with clinical parameters.

In summary, we show that absent expression of WIF1 is due to its high-frequency methylation in CS cell lines and confirmed an aberrant activation of Wnt signaling via WIF1 silencing. High methylation level of WIF1 was associated with both shortened progression-free and overall survival in patients with CS. These data suggest that WIF1 methylation is a promising prognostic predictor in patients with CS. Further study of the mechanisms of WIF1 may provide innovative therapeutic strategies in the treatment of CS focusing on the Wnt signaling pathway.

## Electronic supplementary material

Supplementary Table 1
