# Supplementary material for: Wnt inhibitory factor 1 (WIF1) methylation and its association with clinical prognosis in patients with chondrosarcoma
Source: Sci Rep. 2017 May 8;7:1580. doi: 10.1038/s41598-017-01763-8 (PMC5431504; doi:10.1038/s41598-017-01763-8)
Supplement: Supplementary file 1 — Supplementary Table 1 [file 41598_2017_1763_MOESM1_ESM.docx]

**Title: Wnt inhibitory factor 1 (WIF1) methylation and its association with clinical prognosis in patients with chondrosarcoma**

Pei Liu^1.2^, Jacson K Shen^1^, Francis J Hornicek^1^, Fuyun Liu^2^* and Zhenfeng Duan^1 #^

^1^ Sarcoma Biology Laboratory, Center for Sarcoma and Connective Tissue Oncology, Massachusetts General Hospital, Harvard Medical School, Boston, MA 02114, USA.

^2^Department of Orthopaedics, The Third Affiliated Hospital of Zhengzhou University, Zhengzhou, Henan 450052, People’s Republic of China

Corresponding author: *Fuyun Liu, Department of Orthopaedics, The Third Affiliated Hospital of Zhengzhou University, 7 Kangfu Rd, Zhengzhou, Henan 450052, People’s Republic of China. Phone: 86-13903843598; Fax: 86-371-66902000. E-mail address: liufuyun111@126.com

^#^Zhenfeng Duan, Sarcoma Biology Laboratory, Center for Sarcoma and Connective Tissue Oncology, Massachusetts General Hospital, 100 Blossom St, Jackson 1115, Boston, MA 02114, USA. Phone: 617-724-3144; Fax: 617-726-3883. E-mail address: [zduan@mgh.harvard.edu](mailto:zduan@mgh.harvard.edu)

**Supplementary Figure S1. Multiple exposures of WIF1 in Figure 2**

Three different exposures of WIF1 western blotting were presented respectively in cell lines SW1353 and CS-1. High-contrast is necessary for highlighting the band.

**Supplementary Figure S2. Full-length images of Figure 2**

The origin of these images is in accordance with figure 2 showing western blotting with Wnt5a/b, Dvl2 and LR.

| **Supplementary Table 1. List of 22 genes analyzed in CS cell lines** | | | |  | |
| --- | --- | --- | --- | --- | --- |
| **Gene** | **Function** | **Cancers with methylation** | **Sarcomas with methylation** | **Methylation reported in CS** | **Ref** |
| BRCA1 | DNA repair, apoptosis | Breast Cancer et al. | leiomyosarcoma | N | [^1^](#_ENREF_1)^,^[^2^](#_ENREF_2) |
| CDKN2A | CDK4 inhibitor, control of cell-cycle G1 progression | Prostate cancer, Lung cancer et al. | liposarcoma | Tissue | [^3-6^](#_ENREF_3) |
| DAPK1 | Interact with p53, induce apoptosis | Lymphoma, Lung cancer et al. | osteosarcoma | N | [^7-9^](#_ENREF_7) |
| GSTP1 | Metabolism, detoxification, elimination of genotoxic foreign compounds | Prostate cancer et al. | soft tissue sarcoma | N | [^10^](#_ENREF_10)^,^[^11^](#_ENREF_11) |
| MGMT | DNA repair enzyme | Lung cancer et al. | soft tissue sarcoma, osteosarcoma | N | [^12-14^](#_ENREF_12) |
| PTEN | Cell growth, migration, apoptosis | Esophageal cancer et al. | soft tissue sarcoma | N | [^15^](#_ENREF_15)^,^[^16^](#_ENREF_16) |
| RUNX3 | Cell cycle arrest, apoptosis | Leukemia et al. | chondrosarcoma | Tissue | [^17^](#_ENREF_17)^,^[^18^](#_ENREF_18) |
| TIMP3 | Inhibit tumor growth, angiogenesis, invasion, metastasis | Gastric cancer et al. | osteosarcoma | N | [^8^](#_ENREF_8)^,^[^19^](#_ENREF_19) |
| TP73 | Kinase inhibitor p21^waf1^, induce apoptosis | breast cancer et al. | N | N | [^20^](#_ENREF_20) |
| VHL | Control of angiogenesis and cell cycle | Renal cell carcinomas et al. | N | N | [^21^](#_ENREF_21) |
| APC | Regulator of Wnt signaling in cell migration and adhesion | Prostate cancer, Colon cancer et al. | [liposarcoma](http://www.ncbi.nlm.nih.gov/pubmed/16858687) | N | [^22-24^](#_ENREF_22) |
| CDH1 | Cell adhesion | Colon cancer, Stomach cancer et al. | osteosarcoma | Tissue | [^6^](#_ENREF_6)^,^[^22^](#_ENREF_22)^,^[^25^](#_ENREF_25)^,^[^26^](#_ENREF_26) |
| CDH13 | Reduce invasion and growth | Bladder cancer, Lung cancer et al. | N | N | [^27^](#_ENREF_27)^,^[^28^](#_ENREF_28) |
| FHIT | Cellular differentiation and apoptosis | Breast cancer et al. | chondrosarcoma | Tissue | [^6^](#_ENREF_6)^,^[^29^](#_ENREF_29) |
| MLH1 | DNA repair | Stomach cancer, Lung cancer et al. | soft tissue sarcoma | N | [^30-32^](#_ENREF_30) |
| NEUROG1 | Neuron differentiation | Colorectal cancer et al. | N | N | [^33^](#_ENREF_33) |
| RASSF1 | Cell cycle | Lung cancer et al. | Ewing sarcoma, osteosarcoma | N | [^34-36^](#_ENREF_34) |
| ESR1 | Estrogen receptor, regulation of gene expression | Cervical cancer et al. | N | N | [^37^](#_ENREF_37) |
| SOCS1 | Inhibit cytokine signaling | Hepatocellular carcinoma et al. | N | N | [^38^](#_ENREF_38) |
| WIF1 | Inhibit WNT signaling | Oral and oropharyngeal cancer et al. | osteosarcoma | N | [^39^](#_ENREF_39)^,^[^40^](#_ENREF_40) |
| RARB | Cell growth and differentiation | Breast cancer et al. | N | N | [^41^](#_ENREF_41) |
| PDLIM4 | Bone development | Prostate cancer et al. | N | N | [^42^](#_ENREF_42) |
| N represent "data not available" | | | | |  |

**Reference**

1 Otani, Y. *et al.* BRCA1 promoter methylation of normal breast epithelial cells as a possible precursor for BRCA1-methylated breast cancer. *Cancer science* **105**, 1369-1376, doi:10.1111/cas.12506 (2014).

2 Xing, D. *et al.* A role for BRCA1 in uterine leiomyosarcoma. *Cancer research* **69**, 8231-8235, doi:10.1158/0008-5472.CAN-09-2543 (2009).

3 Bearzatto, A. *et al.* p16(INK4A) Hypermethylation detected by fluorescent methylation-specific PCR in plasmas from non-small cell lung cancer. *Clinical cancer research : an official journal of the American Association for Cancer Research* **8**, 3782-3787 (2002).

4 Davidovic, R. *et al.* p14(ARF) methylation is a common event in the pathogenesis and progression of myxoid and pleomorphic liposarcoma. *Medical oncology* **30**, 682, doi:10.1007/s12032-013-0682-9 (2013).

5 Feng, W., Han, Z., Zhu, R., Liu, P. & Liu, S. Association of p16 gene methylation with prostate cancer risk: a meta-analysis. *Journal of B.U.ON. : official journal of the Balkan Union of Oncology* **20**, 1074-1080 (2015).

6 Ropke, M., Boltze, C., Neumann, H. W., Roessner, A. & Schneider-Stock, R. Genetic and epigenetic alterations in tumor progression in a dedifferentiated chondrosarcoma. *Pathology, research and practice* **199**, 437-444, doi:10.1078/0344-0338-00443 (2003).

7 Giachelia, M. *et al.* Quantification of DAPK1 promoter methylation in bone marrow and peripheral blood as a follicular lymphoma biomarker. *The Journal of molecular diagnostics : JMD* **16**, 467-476, doi:10.1016/j.jmoldx.2014.03.003 (2014).

8 Hou, P. *et al.* Quantitative analysis of promoter hypermethylation in multiple genes in osteosarcoma. *Cancer* **106**, 1602-1609, doi:10.1002/cncr.21762 (2006).

9 Rosell, A. *et al.* Aberrant gene methylation and bronchial dysplasia in high risk lung cancer patients. *Lung cancer* **94**, 102-107, doi:10.1016/j.lungcan.2016.02.003 (2016).

10 Kawaguchi, K. *et al.* DNA hypermethylation status of multiple genes in soft tissue sarcomas. *Modern pathology : an official journal of the United States and Canadian Academy of Pathology, Inc* **19**, 106-114, doi:10.1038/modpathol.3800502 (2006).

11 Maldonado, L. *et al.* GSTP1 promoter methylation is associated with recurrence in early stage prostate cancer. *The Journal of urology* **192**, 1542-1548, doi:10.1016/j.juro.2014.04.082 (2014).

12 Fang, N., Gu, J., Wei, H., You, J. & Zhou, Q. [A meta-analysis of Association between MGMT gene promoter methylation and non-small cell lung cancer]. *Zhongguo fei ai za zhi = Chinese journal of lung cancer* **17**, 601-605, doi:10.3779/j.issn.1009-3419.2014.08.04 (2014).

13 Guo, J. *et al.* Research on DNA methylation of human osteosarcoma cell MGMT and its relationship with cell resistance to alkylating agents. *Biochemistry and cell biology = Biochimie et biologie cellulaire* **91**, 209-213, doi:10.1139/bcb-2012-0100 (2013).

14 Jakob, J. *et al.* O6-methylguanine-DNA methyltransferase (MGMT) promoter methylation is a rare event in soft tissue sarcoma. *Radiation oncology* **7**, 180, doi:10.1186/1748-717X-7-180 (2012).

15 Sun, Z. *et al.* PTEN gene is infrequently hypermethylated in human esophageal squamous cell carcinoma. *Tumour biology : the journal of the International Society for Oncodevelopmental Biology and Medicine* **36**, 5849-5857, doi:10.1007/s13277-015-3256-y (2015).

16 Yin, L. *et al.* Analysis of PTEN methylation patterns in soft tissue sarcomas by MassARRAY spectrometry. *PloS one* **8**, e62971, doi:10.1371/journal.pone.0062971 (2013).

17 Estecio, M. R. *et al.* RUNX3 promoter hypermethylation is frequent in leukaemia cell lines and associated with acute myeloid leukaemia inv(16) subtype. *British journal of haematology* **169**, 344-351, doi:10.1111/bjh.13299 (2015).

18 Jin, Z., Han, Y. X. & Han, X. R. Loss of RUNX3 expression may contribute to poor prognosis in patients with chondrosarcoma. *Journal of molecular histology* **44**, 645-652, doi:10.1007/s10735-013-9511-x (2013).

19 Guan, Z., Zhang, J., Song, S. & Dai, D. Promoter methylation and expression of TIMP3 gene in gastric cancer. *Diagnostic pathology* **8**, 110, doi:10.1186/1746-1596-8-110 (2013).

20 Moelans, C. B. *et al.* Methylation biomarkers for pleomorphic lobular breast cancer - a short report. *Cellular oncology* **38**, 397-405, doi:10.1007/s13402-015-0241-9 (2015).

21 Becket, E. *et al.* Identification of DNA Methylation-Independent Epigenetic Events Underlying Clear Cell Renal Cell Carcinoma. *Cancer research* **76**, 1954-1964, doi:10.1158/0008-5472.CAN-15-2622 (2016).

22 Michailidi, C. *et al.* Expression and promoter methylation status of hMLH1, MGMT, APC, and CDH1 genes in patients with colon adenocarcinoma. *Experimental biology and medicine* **240**, 1599-1605, doi:10.1177/1535370215583800 (2015).

23 Sievers, S. *et al.* Hypermethylation of the APC promoter but lack of APC mutations in myxoid/round-cell liposarcoma. *International journal of cancer* **119**, 2347-2352, doi:10.1002/ijc.22117 (2006).

24 Zhang, W. *et al.* Correlation between the expression of DNMT1, and GSTP1 and APC, and the methylation status of GSTP1 and APC in association with their clinical significance in prostate cancer. *Molecular medicine reports* **12**, 141-146, doi:10.3892/mmr.2015.3402 (2015).

25 Li, X. J., Zhao, Y. & Ren, H. [E-cadherin expression and CDH1 promoter methylation in sporadic and hereditary gastric cancer]. *Nan fang yi ke da xue xue bao = Journal of Southern Medical University* **35**, 125-127 (2015).

26 Mu, X. *et al.* Chick embryo extract demethylates tumor suppressor genes in osteosarcoma cells. *Clinical orthopaedics and related research* **472**, 865-873, doi:10.1007/s11999-013-3104-6 (2014).

27 Chen, F. *et al.* Clinical significance of CDH13 promoter methylation as a biomarker for bladder cancer: a meta-analysis. *BMC urology* **16**, 52, doi:10.1186/s12894-016-0171-5 (2016).

28 Zhong, Y. H., Peng, H., Cheng, H. Z. & Wang, P. Quantitative assessment of the diagnostic role of CDH13 promoter methylation in lung cancer. *Asian Pacific journal of cancer prevention : APJCP* **16**, 1139-1143 (2015).

29 Liu, L. *et al.* Quantitative detection of methylation of FHIT and BRCA1 promoters in the serum of ductal breast cancer patients. *Bio-medical materials and engineering* **26 Suppl 1**, S2217-2222, doi:10.3233/BME-151527 (2015).

30 Kawaguchi, K. *et al.* Microsatellite instability and hMLH1 and hMSH2 expression analysis in soft tissue sarcomas. *Oncology reports* **13**, 241-246 (2005).

31 Li, Y. *et al.* Predictive value of CHFR and MLH1 methylation in human gastric cancer. *Gastric cancer : official journal of the International Gastric Cancer Association and the Japanese Gastric Cancer Association* **18**, 280-287, doi:10.1007/s10120-014-0370-2 (2015).

32 Wu, F., Lu, M., Qu, L., Li, D. Q. & Hu, C. H. DNA methylation of hMLH1 correlates with the clinical response to cisplatin after a surgical resection in Non-small cell lung cancer. *International journal of clinical and experimental pathology* **8**, 5457-5463 (2015).

33 Herbst, A. *et al.* Methylation of NEUROG1 in serum is a sensitive marker for the detection of early colorectal cancer. *The American journal of gastroenterology* **106**, 1110-1118, doi:10.1038/ajg.2011.6 (2011).

34 Lim, S. *et al.* Inactivation of the RASSF1A in osteosarcoma. *Oncology reports* **10**, 897-901 (2003).

35 Avigad, S. *et al.* Aberrant methylation and reduced expression of RASSF1A in Ewing sarcoma. *Pediatric blood & cancer* **53**, 1023-1028, doi:10.1002/pbc.22115 (2009).

36 Wei, H., Fang, N., Guo, L., Wu, Z. & Zhou, Q. [Meta-analysis of the Association between RASSF1A Gene Promoter Methylation and Non-small Cell Lung Cancer]. *Zhongguo fei ai za zhi = Chinese journal of lung cancer* **18**, 443-450, doi:10.3779/j.issn.1009-3419.2015.07.09 (2015).

37 Kirn, V. *et al.* ESR1 promoter methylation in squamous cell cervical cancer. *Anticancer research* **34**, 723-727 (2014).

38 Liu, M., Cui, L. H., Li, C. C. & Zhang, L. Association of APC, GSTP1 and SOCS1 promoter methylation with the risk of hepatocellular carcinoma: a meta-analysis. *European journal of cancer prevention : the official journal of the European Cancer Prevention Organisation* **24**, 470-483, doi:10.1097/CEJ.0000000000000121 (2015).

39 Rubin, E. M. *et al.* Wnt inhibitory factor 1 decreases tumorigenesis and metastasis in osteosarcoma. *Molecular cancer therapeutics* **9**, 731-741, doi:10.1158/1535-7163.MCT-09-0147 (2010).

40 Paluszczak, J. *et al.* The negative regulators of Wnt pathway-DACH1, DKK1, and WIF1 are methylated in oral and oropharyngeal cancer and WIF1 methylation predicts shorter survival. *Tumour biology : the journal of the International Society for Oncodevelopmental Biology and Medicine* **36**, 2855-2861, doi:10.1007/s13277-014-2913-x (2015).

41 Pirouzpanah, S., Taleban, F. A., Mehdipour, P. & Atri, M. Association of folate and other one-carbon related nutrients with hypermethylation status and expression of RARB, BRCA1, and RASSF1A genes in breast cancer patients. *Journal of molecular medicine* **93**, 917-934, doi:10.1007/s00109-015-1268-0 (2015).

42 Vanaja, D. K. *et al.* PDLIM4 repression by hypermethylation as a potential biomarker for prostate cancer. *Clinical cancer research : an official journal of the American Association for Cancer Research* **12**, 1128-1136, doi:10.1158/1078-0432.CCR-05-2072 (2006).
